# Gate-tuned Josephson effect on the surface of a topological insulator

**DOI:** 10.1186/1556-276X-9-515

**Published:** 2014-09-20

**Authors:** Chunxu Bai, Yanling Yang

**Affiliations:** 1 School of Physics, Anyang Normal University, Anyang 455000, People’s Republic of China; 2 SKLSM, Institute of Semiconductors, Chinese Academy of Sciences, Beijing 100083, People’s Republic of China

**Keywords:** Josephson effect, Topological insulator, Furusaki-Tsukada formula

## Abstract

In the study, we investigate the Josephson supercurrent of a superconductor/normal metal/superconductor junction on the surface of a topological insulator, where a gate electrode is attached to the normal metal. It is shown that the Josephson supercurrent not only can be tuned largely by the temperature but also is related to the potential and the length of the weak-link region. Especially, the asymmetry excess critical supercurrent, oscillatory character, and plateau-like structure have been revealed. We except those phenomena that can be observed in the recent experiment.

## Background

Since the pioneering work of Kane and Mele [1], there has been a great deal of theoretical and experimental investigations concerning the exotic new phase of condensate matter-topological insulator (TI) [2-5]. Originally, a TI state (first termed as the quantum spin Hall phase) is prophesied in graphene based on the spin-orbit interaction and time-reversal symmetry [1]. Shortly after TI state was first proposed in a 2-dimensional (2D) graphene, the amazing quantum state was theoretically proposed independently in HgTe quantum wells [6,7] and the alloy Bi_1-*x*_Sb_*x*_ with a special range of *x*[8]. Unlike the weak intrinsic spin-orbit coupling in graphene [9], the amazing TI states have been observed experimentally soon after the theoretical prediction [10,11]. In general, we can first divide TI into two broad classes in a real-space picture: the 2D TI holding a pair of 1D edge states with Dirac-like dispersion and the 3D TI hosting the 2D massless Dirac fermion states on the surface. In the 3D case, the weak TI and strong TI correspond to the even and odd number of Dirac cones on the surface [12-14]. Because the weak TI is adiabatically connected to stacked layers of 2D TI, the strong TI has received a surge of research activities due to the robustness of its surface states as a genuine new state of matter [2-5,12-14].

An intriguing pitch came into the field when a superconductor is proximate to the surface of strong TI. By the proximity effect of superconductor on the surface state, Majorana fermions are predicted to occur [15,16]. The appearance of such Majorana fermions is expected to lead to a number of unusual electronic properties such as zero-bias conductance anomalies [17,18], non-Abelian statistics [19], electron teleportation [20], and so on. It is also interesting that the appearance of such quasiparticles in the surface of strong TI can also provide us with a smoking gun experimental setup to diagnose them by the fractional Josephson effect [21,22]. The Josephson effect describes a phenomenon of supercurrent through a device known as a Josephson junction [23]. It is a two-particle process in which a Cooper pair in one superconductor can across a weak link into the other superconductor without any voltage applied. Recently, Josephson effect on the surface of strong TI has attracted a lot of attention about the peculiar Majorana fermion [12-14,21,22,24-31]. In most of the conditions, it is assumed that the Fermi level is close to the Dirac point. However, the chemical potential of TI does not always certainly reside at the Dirac point from an experimental point of view. Also, in those studies, a linear junction is generally analyzed by the discretized bound states in the superconductor gap (without the consideration of the continuous spectrum above the gap). But for a finite length scale junction, the continuous spectrum begins to play a partial role to the supercurrent. In addition, in those calculations, it is assumed that a ferromagnet lead is sandwiched between the two superconductor leads to exploit Majorana fermion. Besides charming of Majorana fermion, Josephson junction has an important application in quantum-mechanical circuits, such as superconducting quantum interference device, superconducting qubits, and rapid single flux quantum digital electronics, and so on [32]. Thus, it is also an important thing to understand the fundamental properties of the Josephson effect on the surface of TI.

Hence, in this work, we study the Josephson effect through a Josephson junction on the surface of a strong TI where a gate voltage is exerted on the central normal lead with a finite width. Here, we adopt the Furusaki-Tsukada method [33,34], which is applicable to any length of the junction and any potential strength of the weak link. Based on the method, it will allow us to reveal a number of characteristics, such as the dependence of the supercurrents on relevant variables, such as the length between the two superconductor leads, the temperature, the phase bias, and the gate voltage of the central weak-link region.

## Methods

**Figure 1:**
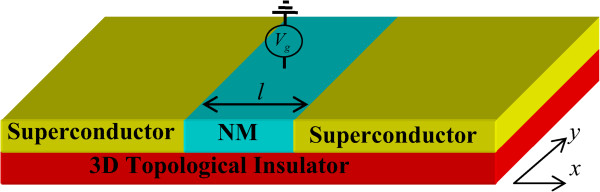
**Schematic diagram of a *S*/ *N */*S *Josephson junction.** The two yellow blocks denote the two s-wave superconductor leads, and the central cyan block denotes a normal metal lead which is deposited on the surface of the 3D topological insulator. By the proximity effect, the s-wave superconducting pair potential is induced in the surface state of the 3D topological insulator. While a gate voltage is applied on the central normal metal lead to tune the Fermi energy.

We consider a ballistic Josephson junction on the surface of TI (see Figure 1) consisting of a normal metal (NM) lead sandwiched by two superconductors. The growth direction is taken along the *x*-axis. The superconductor regions occupy *x* < 0 and *x* > *l*, while the NM region occupies 0 < *x* < *l*. Indeed, the surface state of TI is metallic naturally. However, by means of the proximity effect, a superconductor pair correlation on the surface can be induced in the presence of a superconductor lead [19,30]. Here, the left and right superconductor leads are shown in Figure 1 which denote the two bulk s-wave superconductor leads. As a result, s-wave superconductor pair correlation is induced in topological surface states underneath the superconductor leads. Therefore, the induced singlet superconducting pairing strength can be described by $\Delta \left(x\right)=\Delta \left[e^{i\phi_{L}}\Theta \left(x\right)+e^{i\phi_{R}}\Theta \left(x-l\right)\right]$ where *Θ*(*x*) is the Heaviside step function, *Δ* and *φ* are the superconducting gap and the phase of superconducting order parameter, respectively. The temperature dependence of the bulk pair potential *Δ* is given by the usual formula $\Delta \left(T\right)=\Delta \left(0\right)\tanh\left(1.74\sqrt{T_{C}/T-1}\right)$[35]. On the other hand, the potential in NM can be adjusted by a top-gate lead sketched in Figure 1. Since the zero of potential is arbitrary, we set the potential as *U*(*x*) = -*UΘ*(*x*)*Θ*(*x* - *l*). Notice that the mean-field requirement of superconductivity is satisfied as long as *Δ* < < *E*_*F*_ (*E*_*F*_ is the Fermi energy). Moreover, we also assume that the width of the nanostructure *W* is very big; hence, the details of the microscopic description of the strip edges become irrelevant.

Due to the translational invariance in *y*-direction, the *y*-component of the momentum is conserved and the Hamiltonian (wave function) is reduced to an effective 1D one (only *x*-component). Thus, the low-energy excitation quasiparticle propagation in the ballistic Josephson junction can be described by the following Dirac-Bogoliubov-de Gennes equation [30]:

(1) $$\left(\begin{array}{cc} H-E_{F} & \Delta \left(x\right)\\ \Delta^{*}\left(x\right) & E_{F}-\sigma_{y}H^{*}\sigma_{y} \end{array}\right)\Psi =\mathrm{E\Psi}$$

where $H=\hbar v_{F}\vec{p}\times \vec{\sigma }+U\left(x\right)$ and *v*_*F*_ is the Fermi velocity, $\vec{\sigma }$ is the Pauli matrices, the four-dimensional spinor *Ψ* contains $u=\left(\begin{array}{cc} \psi_{↑}, & \psi_{↓} \end{array}\right)$ for the electron-like quasiparticle and $v=\left(\begin{array}{cc} \psi_{↑}^{*}, & \psi_{↓}^{*} \end{array}\right)$ for the hole-like quasiparticle, and *E* is the quasiparticle energy measured from *E*_*F*_. In the following, we set *ℏ* = *v*_*F*_ = 1.

By solving (1), the wave functions in the superconductor and NM regions can be expressed in a specific form. In the NM, the wave functions are given by

(2) $$\begin{array}{l} \Psi_{N}^{e\pm }=\left(\begin{array}{cccc} 1, & \left(\mathrm{iq}\pm k_{N}^{e}\right)/\left(\epsilon -U+E_{F}\right), & 0, & 0 \end{array}\right)e^{i\left(\pm k_{N}^{e}x+\mathrm{qy}\right)}\\ \Psi_{N}^{h\pm }=\left(\begin{array}{cccc} 0, & 0, & 1, & -\left(\mathrm{iq}\pm k_{N}^{h}\right)/\left(\epsilon +U-E_{F}\right) \end{array}\right)e^{i\left(\pm k_{N}^{h}x+\mathrm{qy}\right)} \end{array}$$

where $\Psi_{N}^{e\pm }$ and $\Psi_{N}^{h\pm }$ are the wave functions traveling along the ± *x* directions with a transverse momentum *q* and an energy *ϵ* for electron and hole, respectively, and $k_{N}^{e}=\sqrt{-q^{2}+{\left(\epsilon -U+E_{F}\right)}^{2}}$ and $k_{N}^{h}=\sqrt{-q^{2}+{\left(\epsilon +U-E_{F}\right)}^{2}}$ are the momentums along the *x*-axis. Note that the evanescent solutions can be included easily in the present case to ensure the current conservation in the calculations. Similarly, in the superconductor leads, the wave functions are

(3) $$\begin{array}{l} \Psi_{S}^{e\pm }=\left(\begin{array}{cccc} u, & u\eta_{\pm }^{e}, & ve^{-i\phi_{\zeta }}, & v\eta_{\pm }^{e}e^{-i\phi_{\zeta }} \end{array}\right)e^{i\left(\pm k_{S}^{e}x+\mathrm{qy}\right)}\\ \Psi_{S}^{h\pm }=\left(\begin{array}{cccc} v, & v\eta_{\pm }^{h}, & ue^{-i\phi_{\zeta }}, & u\eta_{\pm }^{h}e^{-i\phi_{\zeta }} \end{array}\right)e^{i\left(\pm k_{S}^{h}x+\mathrm{qy}\right)}\\ \eta_{\pm }^{e}=\left(\mathrm{iq}\pm k_{S}^{e}\right)/\left(E_{F}+\sqrt{\epsilon^{2}-\Delta^{2}}\right)\\ \eta_{\pm }^{h}=\left(\mathrm{iq}\pm k_{S}^{h}\right)/\left(E_{F}-\sqrt{\epsilon^{2}-\Delta^{2}}\right) \end{array}$$

where $k_{S}^{e\left(h\right)}=\sqrt{-q^{2}-\Delta^{2}+\epsilon^{2}+E_{F}^{2}+\left(-\right)2E_{F}\sqrt{\epsilon^{2}-\Delta^{2}}}$, the coherence factors are given by $u=\sqrt{\left(1+\sqrt{\epsilon^{2}-\Delta^{2}/\epsilon }\right)/2},\;v=\sqrt{\left(1-\sqrt{\epsilon^{2}-\Delta^{2}/\epsilon }\right)/2}\text{,}$ and *ζ* = *L* or *R*.

For an electron-like quasiparticle of energy *E* > *Δ* and transverse momentum *q* (the incident angle *θ*) incident from the left superconductor lead, the corresponding wave functions in the three regions can be written as

(4) $$\begin{array}{l} \Psi_{L}=\Psi_{S}^{e+}+r^{1}\Psi_{S}^{e-}+r_{A}^{1}\Psi_{S}^{h-}\\ \Psi_{M}=f\psi_{N}^{e+}+g\Psi_{N}^{h+}+m\psi_{N}^{e-}+n\Psi_{N}^{h-}\\ \Psi_{R}=t^{1}\Psi_{S}^{e+}+t_{A}^{1}\Psi_{S}^{h+} \end{array}$$

where *r*^1^ and $r_{A}^{1}$ are the amplitudes of normal and Andreev reflections, respectively, *f*, *g*, *m*, and *n* are the corresponding transmission and reflection amplitudes in NM, and *t*^1^ and $t_{A}^{1}$ are the amplitudes of electron-like and hole-like quasiparticles in the right superconductor lead.

For a hole-like quasiparticle incident from the left superconductor lead with energy *E* > *Δ* and transverse momentum *q* (the incident angle *θ*), the corresponding wave functions in the three regions have the following forms:

(5) $$\begin{array}{l} \Psi_{L}=\Psi_{S}^{h+}+r^{2}\Psi_{S}^{h-}+r_{A}^{2}\Psi_{S}^{e-}\\ \Psi_{M}=f^{\prime }\psi_{N}^{e+}+g^{\prime }\Psi_{N}^{h+}+m^{\prime }\psi_{N}^{e-}+n^{\prime }\Psi_{N}^{h-}\\ \Psi_{R}=t^{2}\Psi_{S}^{e+}+t_{A}^{2}\Psi_{S}^{h+} \end{array}$$

Appling the continuity boundary conditions of the wave functions at the boundary *Ψ*_*L*_(0) = *Ψ*_*M*_(0) and *Ψ*_*M*_(*l*) = *Ψ*_*R*_(*l*), the amplitudes $r_{A}^{1}$ and $r_{A}^{2}$ can be obtained directly. As the analytical results for these coefficients are tedious, we only give the numerical results in the following section.

After that, it is straightforward to calculate the dc Josephson current in terms of the Andreev reflection amplitudes by using the temperature Green's function formalism [33,34],

(6) $$I=\frac{\mathrm{e\Delta}}{2\hbar }\sum_{\mathrm{\sigma q}}k_{B}T\sum_{\omega_{n}}\frac{1}{2\Omega_{n}}\left(k_{n}^{e}+k_{n}^{h}\right)\left(\frac{r_{n}^{1}}{k_{n}^{e}}-\frac{r_{n}^{2}}{k_{n}^{h}}\right)$$

where $k_{n}^{e}$, $k_{n}^{h}$, $r_{n}^{1}$, and $r_{n}^{2}$ are obtained from $k_{S}^{e}$, $k_{S}^{h}$, $r_{A}^{1}$, and $r_{A}^{2}$ by the analytic continuation *ϵ* → *iω*_*n*_, the Matsubara frequencies are *ω*_*n*_ = *πk*_*B*_*T*(2*n* + 1) with *n* = 0, ±1, ±2, …, and $\Omega_{n}=\sqrt{\omega_{n}^{2}+\Delta^{2}}$. In effect, most of the existing literatures about Josephson junction on the surface of TI have just focused on the short-junction cases *l* ≪ *ξ* with *ξ* = 1/*Δ* the superconducting coherence length. In particular, the Josephson junctions with a normal metal weak link in [22] are only considered the discretized bound states even in the calculation for large length scales. However, in the long-junction cases *l* ≫ *ξ*, the Andreev level proliferation and the phase dependence of continuous spectrum must be taken into account. By employing the Furusaki and Tsukada formula [33,34], we will provide a theoretical investigation for a finite temperature and an arbitrary length scale. Moreover, going beyond the weak-link junction restriction, in this paper, we will also investigate the tunnel junction case through the Furusaki and Tsukada formula [33,34]. In the Andreev approximation, we can obtain the following form of the dc Josephson current by integrating over *q*,

(7) $$I=\frac{2\pi k_{B}\mathrm{T\Delta}}{\mathrm{eR}}\int_{0}^{\pi /2}\cos\mathrm{\theta d\theta}\sum_{\omega_{n}}\frac{1}{\Omega_{n}}\left(r_{n}^{1}-r_{n}^{2}\right)$$

Note that $R=\frac{2\pi^{2}\hbar^{2}v_{F}}{We^{2}E_{F}}$ where *W* is the width of the junction. Certainly, using (7) the dc Josephson current for the present junction can be obtained easily by the numerical calculations.

## Results and discussion

From an experimental point of view, due to the lattice mismatch between the bulk superconductor and the TI, the induced superconducting gap on the surface state of the TI can be expected to be substantially reduced in magnitude. In general, for a conventional s-wave superconductor such as Al or Nb, the gap and critical temperature can be assumed to *Δ* ~ 0.1 meV and *T*_*C*_ = 23 K, respectively. Here, we estimate the Fermi velocity as *v*_*F*_ ≈ 1 × 10^5^ m/s. The superconducting coherence length is then *ξ* ~ 600 nm. In practice, the bulk band gap of TI opening can be observed on the order of 20 to 300 meV that depends on the material [36]. Moreover, the Fermi energy *E*_*F*_ can be tuned arbitrarily by either using the electric field effect or local chemical doping [37]. Therefore, such a junction with *E*_*F*_ = 10 ~ 10^3^*Δ* can be experimentally achieved within the present-day technique. Meanwhile, the requirement of the transport inside the bulk gap can be fulfilled. Therefore, the parameters and the results in this study are authentic.

**Figure 2:**
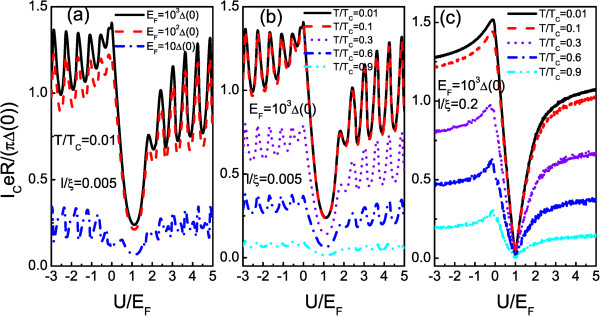
**Plot of critical supercurrent as a function of the potential strengths of NM region. (a)** Plot of critical supercurrent as a function of the potential strengths of NM region for different Fermi energies of the system**.** Solid line, dashed line, and dotted line correspond to the Fermi energy with *E*_*F*_/*Δ*(0) = 10, 10^2^, and 10^3^, respectively. In **(b)** and **(c)**, critical supercurrent as a function of the potential strengths for different temperatures is plotted. The other parameters are shown in the figure.

Let us now first consider the critical supercurrent. A plot of the critical supercurrent as a function of the potential *U*/*E*_*F*_ for different Fermi energy *E*_*F*_/*Δ*(0) has been shown in Figure 2a. The parameters used are shown in the figure. From Figure 2a, we can find five noteworthy features. First, it exhibits oscillatory behavior due to the coherent interface effect of the quasiparticles in the central weak-link region. Physically, the Feynman paths of the transmission coefficients must contain the terms such as $\exp\left(\pm ik_{N}^{e}l\right)$ and $\exp\left(\pm ik_{N}^{h}l\right)$, which are the phases acquired by the electron and hole traveling a distance *l*, respectively. Thus, the oscillation period is determined by the resonant condition $\left(k_{N}^{e}-k_{N}^{h}\right)l\cos\left(\theta \right)=2\mathrm{n\pi}$ with $k_{N}^{e\left(h\right)}$ the wave vector in the NM region and *n* an integer. As a consequence, the Andreev bound states are sensitive to the potential *U*/*E*_*F*_ of the middle region, which results in a series of resonance peaks as a function of *U*/*E*_*F*_. Second, the maximum of critical supercurrent reaches an excess value independent of the barrier strength *U*. This can be intuitively elucidated by the fact that a Fermi surface mismatch acts as an effective barrier for the electrons knocking on the interface [38]. However, the effective barrier reaches a saturation value for a large Fermi surface mismatch between the superconductor and the NM regions. As a consequence, the advent of effective excess barrier leads to the excess character for the maximum of critical supercurrent. Third, critical supercurrent reaches its minimum nonzero value when the chemical potential of the NM is precisely at the Dirac point. In effect *U*/*E*_*F*_ = 1, there are no propagating modes available in NM. Naively, one would expect the critical supercurrent decays to a negligible value. However, in contrast to a conventional material, the semimetal NM behaves as a disordered metal which makes the critical supercurrent survival even with a considerable value [29]. Fourth, the excess critical supercurrent for a negative *U* - *E*_*F*_ is larger than that for a positive *U* - *E*_*F*_. Such feature is a result of the quasiparticle types involved in those two cases. As shown in graphene, the transmission asymmetry has been revealed in the two cases of Klein tunneling and classical motion for a bipolar junction [39]. For a negative *U* - *E*_*F*_, the quasiparticles (both electron-like and hole-like) in NM all transmit in the conduct band. Meanwhile, the quasiparticles in superconductor origin from the same conduct band. As a result, the quasiparticle conversion between NM (Andreev bound states) and superconductor (Cooper pairs) can be served as a classical motion, at least from a point of view of the transmission. For a positive *U* - *E*_*F*_, in contrast, those supercurrent carrying quasiparticles in NM come form the valence band. Thus, the quasiparticles conversion between NM (in the valence band) and superconductor (in the conduct band) origins form the different bands amounting to a Klein tunneling (a superconduting Klein tunneling). Mathematically, the wave functions $\Psi_{N}^{e\pm }$ and $\Psi_{N}^{h\pm }$ in NM are given by (2). From them, we can clearly see that they have a close relationship with *U* - *E*_*F*_. More specifically, the sign of the second component of $\Psi_{N}^{e\pm }$ and the fourth component of $\Psi_{N}^{h\pm }$ is a direct result of the value of *U* - *E*_*F*_. Therefore, the different quasiparticle types in NM will result in a distinct strength of the excess critical supercurrent. Besides, in Figure 2a, the critical supercurrents for *E*_*F*_/*Δ*(0) = 10, 10^2^, and 10^3^ are calculated. As shown in Figure 2a, the Fermi energy of the system plays an important role in the coherent tunneling. Besides the similar oscillatory characters, the important feature revealed is that the critical supercurrent strength increases with the ratio of *E*_*F*_/*Δ*(0).

In Figure 2b,c, we show the dependence of critical supercurrent on potential *U*/*E*_*F*_ for different temperatures with *E*_*F*_/*Δ*(0) = 10^3^. The other parameters are shown in the figure. Figure 2b,c shows the calculated results of critical supercurrent for cases of *l*/*ξ* = 0.005 and *l*/*ξ* = 0.2, respectively. It can be seen clearly that both of them exhibit the monotonic decay feature with increasing temperature *T*. This is due to the thermal effect on Andreev bound states, which tends to reduce Andreev levels in the superconductor gap with increasing temperature *T*. The less number of Andreev levels contributing to the supercurrent thus leads to the suppressed feature. Besides the similar features, it is worthwhile to note that it also exhibits some different characters between the cases of *l*/*ξ* = 0.005 and *l*/*ξ* = 0.2. First, the minimum of critical supercurrent is shown as a decay function of *T*, while it remains nearly constant when the chemical potential of NM is at the Dirac point. Second, it is found that oscillation amplitude of critical supercurrent will disappear with increasing *l*. The physical origin for those phenomena can be given as follows. In central NM well which is formed by the two superconductor leads, the electron-like and hole-like quasiparticles inside the two interfaces will coherently interfere with each other which results in the formation of Andreev bound states. Through those Andreev levels, critical supercurrent will exhibit an oscillation feature for a short junction (*l*/*ξ* = 0.005). On the other hand, for a long junction (*l*/*ξ* = 0.2), as the interference effect decays in NM well, the present structure degenerates into a single junction case and then leads to the disappearance of the oscillation feature. In particular, the decay effect of interference exhibits much remarkable for the case of the evanescent mode (chemical potential of NM at the Dirac point). Thus, the minimum of critical supercurrent remains nearly constant.

**Figure 3:**
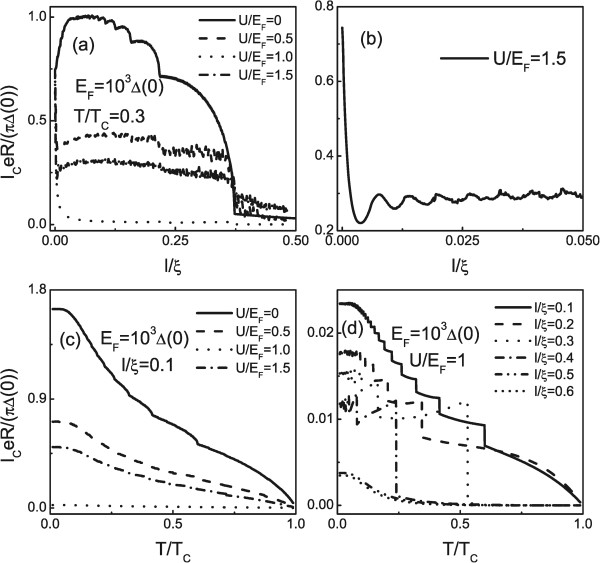
**Plot of the length dependence and temperature dependence of the critical supercurrent. (a)** and **(b)** Plot of the length dependence of the critical supercurrent. **(c)** and **(d)** Plot of the temperature dependence of the critical supercurrent. The parameters are shown in the figure.

Consider now the critical supercurrent as a function of *l*/*ξ* for *E*_*F*_ = 10^3^*Δ*(0) and *T*/*T*_*C*_ = 0.3, at four different potentials *U*/*E*_*F*_ = 0, 0.5, 1, and 1.5, as shown in Figure 3a. The other parameters are similar to the Figure 2. For the case of no Fermi wave vector mismatch between the superconductor and NM (*U* = 0), it is intriguing to note that as the length of the NM increases, the critical supercurrent does not decrease smoothly but shows a plateau-like behavior (see the solid line in Figure 3a). This is a qualitatively new feature as compared to the results of [29] and also contrast to the case of graphene [40]. In order to understand the plateau-like behavior, we repay a close attention to the coherent interface effect in NM. It is important to note that the resonant condition requires a large interval of *l* which reflects a decay effect of the wave feature. Whenever the length of the NM reaches the period condition, the critical supercurrent thus jumps from one step to another adjacent step. Physically, the distinct phenomenon between the present structure and graphene can be ascribed to the different definitions of chirality in the two materials. That is to say, in contrast to graphene where the chirality couples the momentum and the pseudospin degree of freedom, the chirality in the present structure relates the physical spin to the momentum. A nonzero *U* leads to a Fermi wave vector mismatch which amounts to an effective barrier. With the increase of the absolute value of *U*, the coherent interface effect and the decay effect are strengthened. The competition of them makes the critical supercurrent becomes small, but the oscillatory amplitude becomes large as shown in Figure 3a. The reason is that the increase of the Fermi wave vector mismatch with the potential strength results in the increase of quasiparticle interferences in NM. In contrast, the decrease of the amplitude of Andreev reflection reduces the critical supercurrent. In effect, the oscillatory behavior can be seen more clearly from Figure 3b only for the case of *U*/*E*_*F*_ = 1.5.

We now proceed to investigate the temperature dependence of the critical supercurrent in Figure 3c,d. The parameters are shown in the figure. It is shown that the critical supercurrent can be modulated largely by the temperature *T* and a plateau-like structure can be yielded. In particular, the plateau-like behavior may be achieved even when the chemical potential of the NM is precisely at the Dirac point. However, the plateau-like structure washes out for a long junction. The plateau-like structure disappearance behavior can be explained by considering the decay of the quasiparticles interference effect in the NM. Although the plateau-like structure tuned by the temperature *T* is very similar to the case of the length of NM *l*, they have different physical origins. The novel behaviors now can be elucidated as follows. Remember that the temperature dependence of *Δ* is given by $\Delta \left(T\right)=\Delta \left(0\right)\tanh\left(1.74\sqrt{T_{C}/T-1}\right)$. As a result, the critical supercurrent decreases with increasing temperature because the number of Andreev levels (within the superconductor gap) which contributes to the critical supercurrent decreases with increasing temperature. Especially, the interplay between the restriction in the number of the Andreev levels and thermal average of all Andreev levels results in the plateau-like structure.

**Figure 4:**
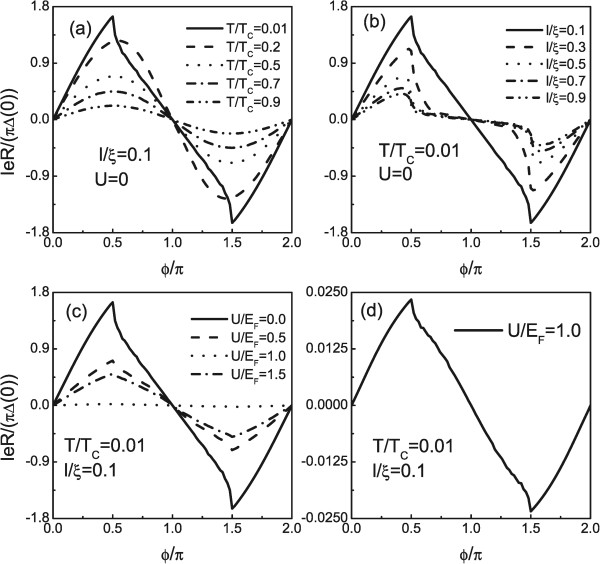
**Plot of the current-phase relationship for different temperatures.***T***(a)**, length *l***(b)**, and potential strength *U***(c)**, **(d)** current-phase relationship for potential strength *U*/*E*_*F*_ = 1. The parameters are shown in the figure.

In the following, we want to show how the fate of the equilibrium current-phase relation depends on the temperature *T*, the length *l*, and the potential strength *U*, as shown in Figure 4a,b,c. The parameters are shown in the figure. In general, a normal incident mode results in an anomalous 4*π* periodic Josephson effect [29,30]. However, the other channels always lead to the 2*π* periodic equilibrium supercurrent. Hence, the 2*π* periodic character dominates the angle-averaged supercurrent and therefore, this supercurrent exhibits a 2*π* period in measurement, as shown in Figure 4. In Figure 4a, we see that for larger temperature current-phase relation has a more sinusoidal shape. For small temperature, there is a sharp peak at *ϕ* = (2*n* + 1)*π*/2 with *n* = 0, ±1, …. Upon increasing *l*, the peaks of equilibrium supercurrents at fixed *T* and *U* are shown by a large suppress. While it is clearly seen that a nonsinusoidal shape current-phase relation can be kept at *ϕ* = (2*n* + 1)*π*/2. Also, for a larger *l*, the sharpness of the peaks becomes less steep which can be ascribed to decay effect of quasiparticles. Finally, we intend to investigate how the current-phase relation depends on *U* at *T*/*T*_*C*_ = 0.01 and *l*/*ξ* = 0.1. The result is shown in Figure 4c,d. The striking feature is that the current-phase relation for different *U* exhibits a similar behavior even at *U*/*E*_*F*_ = 1. To see this phenomenon more clearly, we have only plotted the current-phase relation for *U*/*E*_*F*_ = 1 in Figure 4d. Quantitatively, the magnitude of the supercurrent decreases with decreasing chemical potential in NM since there are few propagating modes available.

## Conclusions

To conclude, we have shown that the Josephson supercurrent not only can be tuned largely by the temperature but also is related to the potential and the length of the weak-link region. Compared to the results that have been obtained, there are some pronounced deviations revealed. Based on the Furusaki and Tsukada formula adopted here where both discretized bound states and the continuum spectrum are included, we can expect that our findings will shed more light on the details of the Josephson supercurrent. With the rapid experimental advance in TI, we can suppose a very efficient Josephson device should be realized in the near future.

## Competing interests

The authors declare that they have no competing interests.

## Authors’ contributions

CB proposed the idea and presided over the study. YY conceived and calculated the setup. CB wrote the paper. Both authors read and approved the final manuscript.
